# Quality of life of Sudanese patients attending a fertility clinic: a mixed methods study

**DOI:** 10.1080/21642850.2021.2007773

**Published:** 2021-12-01

**Authors:** Rasha R. Bayoumi, Emily Koert, Jacky Boivin, Kasisomayajula Viswanath, Margaret McConnell

**Affiliations:** a School of Psychology, University of Birmingham Dubai, Dubai International Academic City, Dubai, UAE; b Department of Educational and Counselling Psychology and Special Education, University of British Columbia, Vancouver, Canada; c Cardiff Fertility Studies Research Group, School of Psychology, Cardiff University, Cardiff, UK; d Department of Social and Behavioral Sciences, Harvard T. H. Chan School of Public Health, Boston, MA, USA; e Department of Global Health and Population, Harvard T. H. Chan School of Public Health, Boston, MA, USA

**Keywords:** Infertility, mixed methods, Sudan, quality of life, FertiQoL

## Abstract

**Background:**

Infertility affects over 50 million people globally, the burden is disproportionately borne by women, especially in low and middle-income countries (LMIC). The impact of infertility on quality of life (QoL) has not been well documented or assessed qualitatively in LMIC like Sudan, where infertility is a pervasive problem. Therefore, the purpose of this mixed-methods study was to assess the fertility-related QoL of infertile individuals in Sudan using the fertility quality of life (FertiQoL) tool.

**Methods:**

We used explanatory sequential design (surveys and interviews) in a fertility clinic in Sudan (January 2017–May 2018). We collected socio-demographic information, medical/reproductive history and used Arabic FertiQoL. We generated descriptive statistics of FertiQoL (core, domain) scores and independent variables; multiple linear regression models to assess the relationship between FertiQoL and dependent variables; and *t*-tests to compare mean core/domain scores. We conducted thematic analysis on qualitative data about the subjective experience of being infertile.

**Results:**

The study included 102 participants (72 women), 70 educated beyond secondary school, mean age 33.89 years (SD = 7.82) and mean duration of infertility was 4.03 years (SD 3.29). Mean FertiQoL core score 76.02 (SD = 16.26), domain scores: emotional 71.61 (SD = 22.04), relational 78.06 (SD = 16.62), mind/body 74.06 (SD 22.53) and social 78.88 (SD = 18.24). Men had better fertility-related QoL.

**Four themes emerged:**

A sense of something missing because of childlessness; social pressure from peoples’ questions; impact on the spousal relationship (which differed amongst participants) and coping (faith-based and non-faith-based) which was necessary when the lived experience led to internal distress.

**Conclusions:**

Infertility negatively impacted the QoL of participants in this study, and women were worse off. Cognitive appraisal, social support and pressure may be key factors influencing the QoL of infertile individuals, therefore they should be encouraged to seek social and professional support. FertiQoL is a useful tool to assess fertility QoL in LMIC like Sudan.

List of abbreviations
ELM: Elaboration likelihood model
IDIs: In-depth interviews
LMIC: Low and middle-income country
OLS: Ordinary linear squares
PT: Patient
QoL: Quality of life
RH: Reproductive Health
FertiQoL: The fertility quality of life tool
RRB: The first author and lead investigator
WHO: World Health Organization
WHOQOL: World Health Organization quality of life assessment tool

## Introduction

Infertility is a health concern that affects over 50 million people globally (World Health Organization, 2018) and the World Health Organization (WHO) estimates that more than 10% of women globally remain childless after 5 years of unprotected sexual intercourse (World Health Organization, 2018). The importance of allocating resources to research and treatment of infertility to ensure adequate knowledge, equity, and accessibility has been indicated and reinforced by the global community (Center for Disease Control and Prevention, 2014; UNFPA, 2004, 2014; United Nations, 1994). Despite gains in areas of reproductive, maternal and new-born health since the millennium development goals, similar changes have not occurred for infertility as indicated by the unchanged prevalence of infertility (Mascarenhas, Flaxman, Boerma, Vanderpoel, & Stevens, 2012). Globally, childlessness has severe negative psychosocial consequences such as depression, anxiety, social isolation, family instability, divorce and intimate partner violence (Dyer, Lombard, & Van Der Spuy, 2009; Greil, Slauson-Blevins, & McQuillan, 2010; Riessman, 2000; Rouchou, 2013). Moreover, in low and middle-income countries (LMIC) the physical, emotional and financial burden is often borne by women (Greil et al., 2010; Riessman, 2000; Rouchou, 2013).

Quality of life (QoL) is a measure of a person’s perception of their life as it relates to goals, expectations, standards, and concerns within the context of culture and values (World Health Organization, 1995). It is an important measure of personal perception of adjustment and has been used to assess the impact of life events and health-related issues. Tools that measure QoL provide a more generalized evaluation of adjustment than tools for mental illness like depression or anxiety inventories. Mental illness tools reflect clinically diagnosable problems, thus might not include people who do not have a mental illness but are none the less suffering. Infertility has been reported to impact QoL negatively (Aarts, Huppelschoten et al., 2011, Aarts, Van Empel et al., 2011; Boivin, Takefman, & Braverman, 2011; Domar, Gross, Rooney, & Boivin, 2015; Huppelschoten et al., 2013; Kitchen, Aldhouse, Trigg, Palencia, & Mitchell, 2017), leading to the development of the fertility quality of life (FertiQoL) tool, which provides a standardized measurement of this impact (Boivin et al., 2011).

### Fertiqol

The FertiQoL is a 26-item questionnaire with core and treatment modules. It has been shown to be a reliable and sensitive tool used to measure fertility-related QoL (Boivin et al., 2011). FertiQoL can aid in the identification of individuals requiring psychosocial support and the specific areas of intervention that should be targeted (Boivin et al., 2011). It has been translated to 48 languages and validated in different samples (Koert, Takefman, & Boivin, 2019). A review of published studies on FertiQoL, reported that there were 41 studies, with 35 independent samples, 16,315 participants from 23 countries (Koert et al., 2019). The range of FertiQoL mean core scores in these 41 studies varied markedly, lowest mean (India) 42.1 and the highest mean (Hungary) 91.7 (Koert et al., 2019). FertiQoL core scores were found to correspond closely with standardized depression and anxiety measures (Aarts, Huppelschoten et al., 2011; Aarts, Van Empel et al., 2011; Dural et al., 2016; Sut & Kaplan, 2015). Lower FertiQoL scores were associated with more time spent reflecting on infertility treatment (Cusatis et al., 2019), with longer duration of infertility and for people who have psychological vulnerability (Koert et al., 2019). Higher FertiQoL scores were associated with higher education level, using ‘Problem Solving Coping strategy’ (Zurlo, Della Volta, & Vallone, 2018) and receiving patient-centred fertility care (Aarts, Huppelschoten et al., 2011; Aarts, Van Empel et al., 2011). The FertiQoL has been used to assess fertility-related QoL globally including the Middle East (Dural et al., 2016; Goker, Yanikkerem, Birge, & Kuscu, 2018; Maroufizadeh, Ghaheri, & Samani, 2017; Sexty et al., 2016; Sut & Kaplan, 2015), but its use in Africa has only been reported once (Patel, van Balen, & Dyer, 2013), and it has never been studied qualitatively.

### Sudan

Sudan is an LMIC with a population of approximately 40 million. Infertility was estimated to be as low as 3%, calculated from demographic health survey data (Larsen, 2000) and primary infertility in clinical samples as high as 80%, 69% and 60%, reported respectively (Abdalla, 2011; Elhussein, Ahmed, Suliman, Yahya, & Adam, 2019; Hussein, Gafoor, Gadir, & Hamad, 2019; Osman, 2011). The importance of infertility has been reflected by the growing number of private treatment clinics and the Sudan Federal Ministry of Health (FMoH) included infertility as one of the products in the new Reproductive Health Strategy (Sudan Federal Ministry of Health, 2017). However, this increased interest has not been matched in research on the topic, with only a handful of scientific publications about infertility in Sudan. Additionally, there are shortcomings in the type and quality of services available such as minimal specialized training, limited privacy and counselling (Khalifa & Ahmed, 2012). In Sudan, there are strong gender norms regarding reproductive health that place the blame of infertility and the burden of help-seeking on women, who also bear the social stigma of childlessness and are obligated to accept divorce or polygamy because of infertility (Al Safi, 2007; Khalifa & Ahmed, 2012). These negative consequences and systemic shortcomings emphasize the importance of addressing infertility and its sequela in Sudan and other LMIC that share similar gender norms and systemic problems. One way to address the negative consequence of infertility in Sudan is through an assessment of fertility-related QoL. Therefore, in this study, we will focus on understanding the impact of being infertile in Sudanese women and men as captured by the FertiQoL and how that compares to their subjective experience as captured by in-depth interviews (IDIs).

The aim of this study was to investigate the effect of infertility on QoL including emotional, relational, social and physical well-being in infertile patients attending a fertility clinic in Sudan. We used a mixed-methods approach and set out three main objectives to achieve this aim. The first objective was to determine the impact of infertility on QoL, overall and on specific domains of QoL (emotional, relational, social and mind/body) as measured by the FertiQoL and to identify health and social determinants (reproductive, cultural, and socio-demographic factors e.g. duration of infertility, consanguinity, age, education) that may be associated with fertility-related QoL (quantitative assessment). The second objective was to examine the nature of the subjective experience of being infertile as reported by the participants in the interviews (qualitative assessment). The third objective was to investigate whether and how the reported lived experience of infertility can explain the associations between the FertiQoL scores and the health and social determinants through triangulation of the data.

## Materials and methods

### Study design

We conducted a mixed-methods study using an explanatory sequential design to determine the impact of being infertile on the QoL of Sudanese patients attending a semi-private fertility clinic in Khartoum (the capital of Sudan). This clinic is based inside a university hospital and payment is subsidized based on patient income, and all patients at the clinic are infertile (general obstetrical and gynecologically cases are seen elsewhere). We collected quantitative data through a survey of the participants using the FertiQoL tool. We conducted semi-structured IDIs with a sub-sample from the same clinic. IDIs were used to ascertain the subjective lived experience of being infertile in Sudan to help explain the survey results in more depth. No financial incentives were offered to participants.

### Participants and recruitment

We used convenience sampling to recruit patients (women and men) attending the clinic from January 2017 to May 2018. There were no exclusion criteria. We approached patients (individuals not couples) in the waiting room and invited them to participate in the study. Recruitment for the IDIs continued until saturation of data was reached, and there was data replication and redundancy (no new themes/new perspectives on themes), the point of diminishing returns was reached (Bowen, 2008).

### Procedure

Patients who agreed to participate were taken to a private room where they were briefed about the study and signed the consent form. All communication with participants was in Arabic and all participants completed the Arabic FertiQoL. For the first group (Group A), research assistants handed out hardcopies of the materials (background information form and Arabic FertiQoL) that participants filled out independently. Research assistants were available for clarification, collected materials, ensured all items were completed, thanked and debriefed the participants.

RRB conducted, and audio recorded the IDIs (approximately 30 min) with the second group (Group B). During the IDIs, participants were asked the background information form questions, they were asked how infertility had impacted their lives and which areas were most impacted, followed by verbal administration of Arabic FertiQoL. RRB scored FertiQoL using the online version of the tool (www.fertistat.com), and shared scores (including visual representation/bar chart) with participants and asked them to discuss how they felt about their results and whether the results reflected their subjective experience of being infertile. All participants were thanked and debriefed.

### Materials

Materials included a consent form (including briefing), a background information form, Arabic FertiQoL, debriefing and an interview topic guide for Group B, see supplemental materials. We used the background information form to ascertain demographic (e.g. gender and age), medical (e.g. diabetes, thyroid illnesses) and reproductive history (e.g. duration of infertility, number of previous spontaneous pregnancies).

We used the Arabic FertiQoL core module (www.fertiqol.com) to provide a quantitative measurement of the impact of infertility on QoL. The core module is used to evaluate the impact of infertility on four areas of life: emotional, mind/body, relational and social domains (Boivin et al., 2011), it includes two questions about overall physical health and QoL satisfaction and six questions for each domain. The emotional domain questions relate to feelings and coping with fertility problems, the mind/body domain questions relate to physiological impact (e.g. fatigue), the relational domain examines the impact of fertility problems on the relationship between the participant and his/her partner, and the social domain assesses the impact on social relationships (Koert et al., 2019). The core module is scored out of 100 with lower scores indicating more negative impact on QoL and higher scores indicating less impact, however, there is no consensuses about a specific cut-off (Koert et al., 2019).

We used the interview topic guide open-ended questions to elicit the lived experience of being infertile (e.g. How has infertility impacted your life? What are the important areas of your life that infertility has impacted positively or negatively?).

### Translation

RRB, in collaboration with local fertility experts in Sudan, translated the materials and interview transcripts (RRB transcribed the interviews). An independent research assistant conducted back-translation of relevant quotes to ensure translation accuracy.

### Reflexive statement

The research team included two experienced qualitative researchers with content expertize in infertility. RRB a clinical psychologist and researcher, familiar with the cultural context (Sudanese heritage and experience living in the country), brought insights to the data from the perspective of an insider. RRB participated in study design and preparing interview materials and conducted the interviews. EK is a psychologist who has worked in the field of infertility for the past 12 years and has worked with FertiQoL in different populations. She brings insights about the phenomena and about the impact of infertility on QoL in different populations. As a non-Sudanese, EK had an outsider view of the culture. RRB and EK jointly conducted qualitative data analysis, interpretation of results and manuscript preparation. The awareness of our research positions in relation to the content-led to rich discussions during analysis.

### Data analysis

#### Quantitative analysis

First, we outlined the conceptual framework which illustrated that socio-demographic, cultural and reproductive factors would be associated with the FertiQoL scores. Then we conducted quantitative analysis, using STATA (version 15) to generate descriptive statistics, multiple regressions and *t*-tests.

To address the first objective, we calculated means core and domain scores. We conducted two sample *t*-tests to compare the means for core and domain scores, to identify which areas of QoL (domain) were most impacted by infertility. We used multiple linear regression, ordinary linear squares (OLS) to identify health and social determinants that maybe associated with fertility-related QoL (FertiQoL core and domain scores). The multiple linear regression model included the following socio-demographic, reproductive and cultural variables: age (greater/less than 35), gender (woman/man), living (urban/rural), education (greater/less than secondary school education), duration of infertility (greater/less than one year), cause of infertility (female factor only/other causes [male factor only, both, unknown and undiagnosed]) and consanguinity (married to blood relative).

#### Qualitative thematic analyses of in-depth interviews

To address the second objective, we used qualitative thematic analysis to examine the nature of the subjective experience of being infertile. We used inductive coding to analyse the transcribed qualitative data (Braun & Clarke, 2006). Inductive coding is a qualitative coding method that is data-driven without any analytic preconception. The researcher interprets the data without a pre-existing framework and the themes that emerge from the raw data are used to develop a conceptual model (Braun & Clarke, 2006). Using inductive coding, both researchers coded the whole data set and compared codes, RRB coded transcripts 1–10 first and EK coded 11–20 first. Each coder derived initial codes from interview data for those participants and discussed the meaning of codes through analytic process memos. Disagreements were resolved through discussions until consensus was reached. Coders discussed preliminary thematic groupings of codes to deepen the analytic process to ensure cohesiveness of each theme and consistency with the overall meanings in the dataset. Coders documented the thematic analysis process including analytic process memos and reflective notes creating an audit trail. To ensure trustworthiness of the findings, the data collection and analysis was guided by best practice guidelines for qualitative research in the Critical Appraisal Skills Programme (Critical Appraisal Skills Programme, 2014) and Meyrick (Meyrick, 2006).

To address the third objective, we used mixed methods to investigate whether and how the reported lived experience of infertility (qualitative data from the IDIs) can explain the quantitative associations between the FertiQoL (core and domain) scores and the health and social determinants. To achieve this, we conducted a second round of thematic analysis to triangulate the data. Triangulation is the use of multiple data sources or multiple methods to enhance the understanding of a phenomenon (Patton, 1999). We compared the qualitative themes with the quantitative descriptive statistics for variables such as age, duration and type of infertility to see if they corresponded. We also examined the qualitative data to help explain the results of the regression analysis.

### Ethics approval and consent to participate

Ethical approval was sought and provided by the Department of Obstetrics and Gynaecology, and the Department of Psychology, at the respective institutions. Participants signed consent forms after being briefed about the study.

## Results

### Socio-demographic, cultural and reproductive characteristics

**Figure 1.**
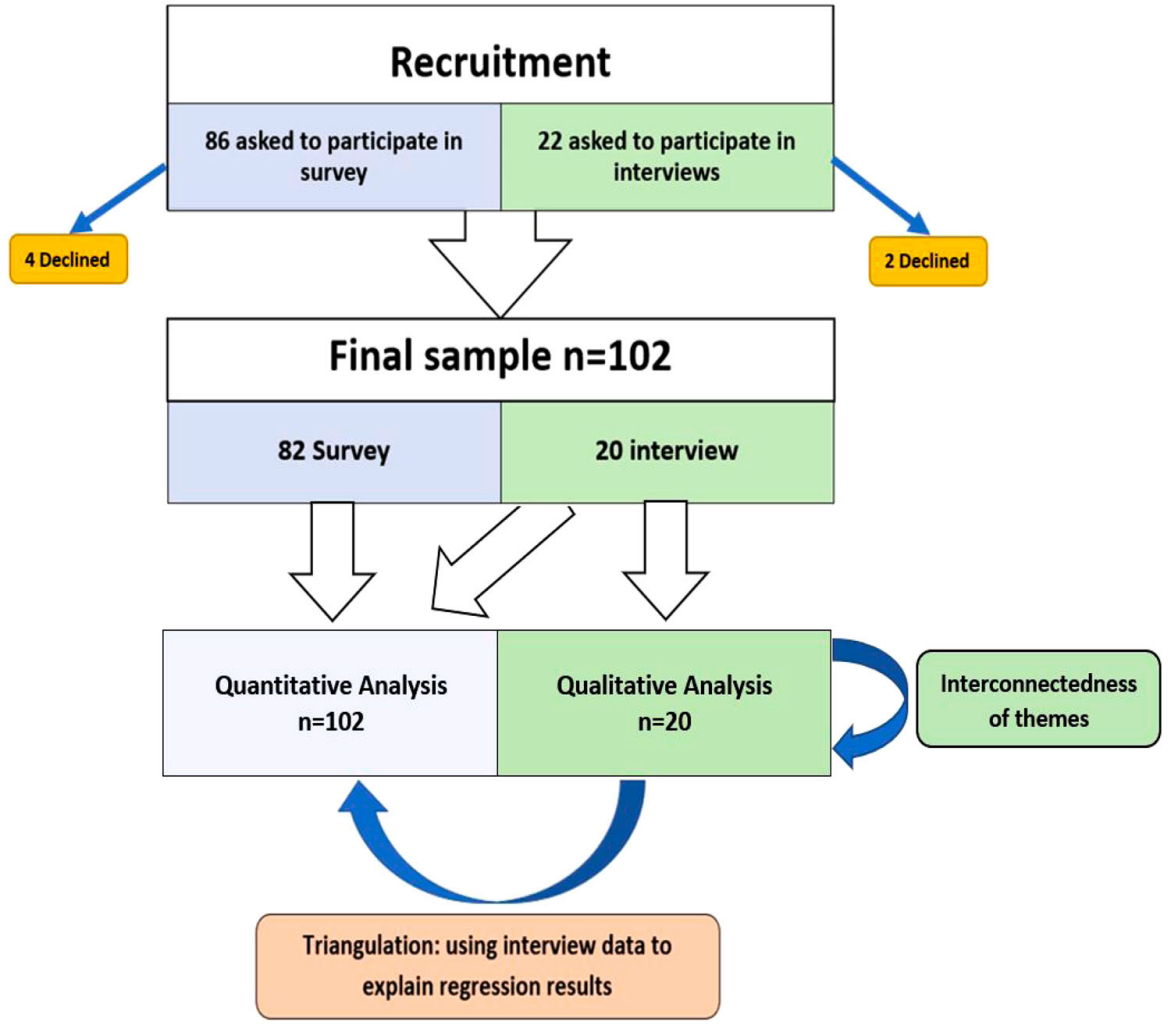
Flowchart demonstrating the recruitment and analysis processes used in the study. This figure shows that 86 patients were approached to participate in the survey, of whom 4 declined and 82 completed the survey. The figure also shows that 22 patients were approached to participate in the interview, of whom 2 decline and 20 completed the interviews. Quantitative analysis was conducted for the whole sample, *n* = 102 and qualitative analysis was conducted for the interview sample, *n* = 20. Qualitative analysis led to an understanding of the interconnectedness of the themes that emerged. Mixed methods analysis like triangulation, used interview data to explain results of quantitative analysis (regression).

The flowchart in Figure 1 shows the recruitment and analysis processes. Of the 108 patients approached to participate, six declined (all women), and 102 completed the study, 82 completed the survey only and 20 (17 women and 3 men) participated in interviews (qualitative sample). Of the 102 participants, 72 (70.6%) were women and thirty (29.4%) were men, see Table 1. The majority were educated beyond secondary school (70, 68.6%) and lived in urban areas (73, 71.6%). The mean age was 33.89 (SD = 7.82, range 17–62) years and the mean duration of infertility was 4 years (SD = 3.29, range 1 month to 14 years), see Table 1.

**Table 1.** Sample characteristics of the study population, including demographic, medical and reproductive history.

Characteristic		Mean (SD)
Age	All	33.89 (7.82) years
	Women	31.96 (6.36) years
	Men	38.57 (8.94) years
Duration of marriage^a^		4.62 (3.46) years
Duration of infertility		4.03 (3.29) years
Gender (*N* = 102)	Women	70.6
	Men	29.4
Principal residence	Urban	71.6
	Rural	28.4
Highest achieved education	Secondary or less	31.4
	More than secondary	68.6
Consanguineous marriage	YES	52.9
	NO	47.1
*Female genital mutilation (*N* = 58^b^)	YES	96.6
	NO	3.4
Source of infertility^c^	Female factor only	34.3
	All other sources	65.7
Previous pregnancy (*N* = 73^d^)	YES	30.6
	NO	69.4

Note: SD = standard deviation; *N* = sample size; *N* = 102 unless otherwise specified.

^a^ All participants were married at the time of the study but not necessarily presented at the clinic with their spouse.

^b^ Only 58 women were asked if they had undergone Female Genital Mutilation.

^c^ Self-reported by the participant, when asked what diagnosis of infertility they had received.

^d^ Only women participants were asked about previous pregnancies.

### Objective 1: FertiQoL scores and associated factors

#### The FertiQoL scores

**Figure 2.**
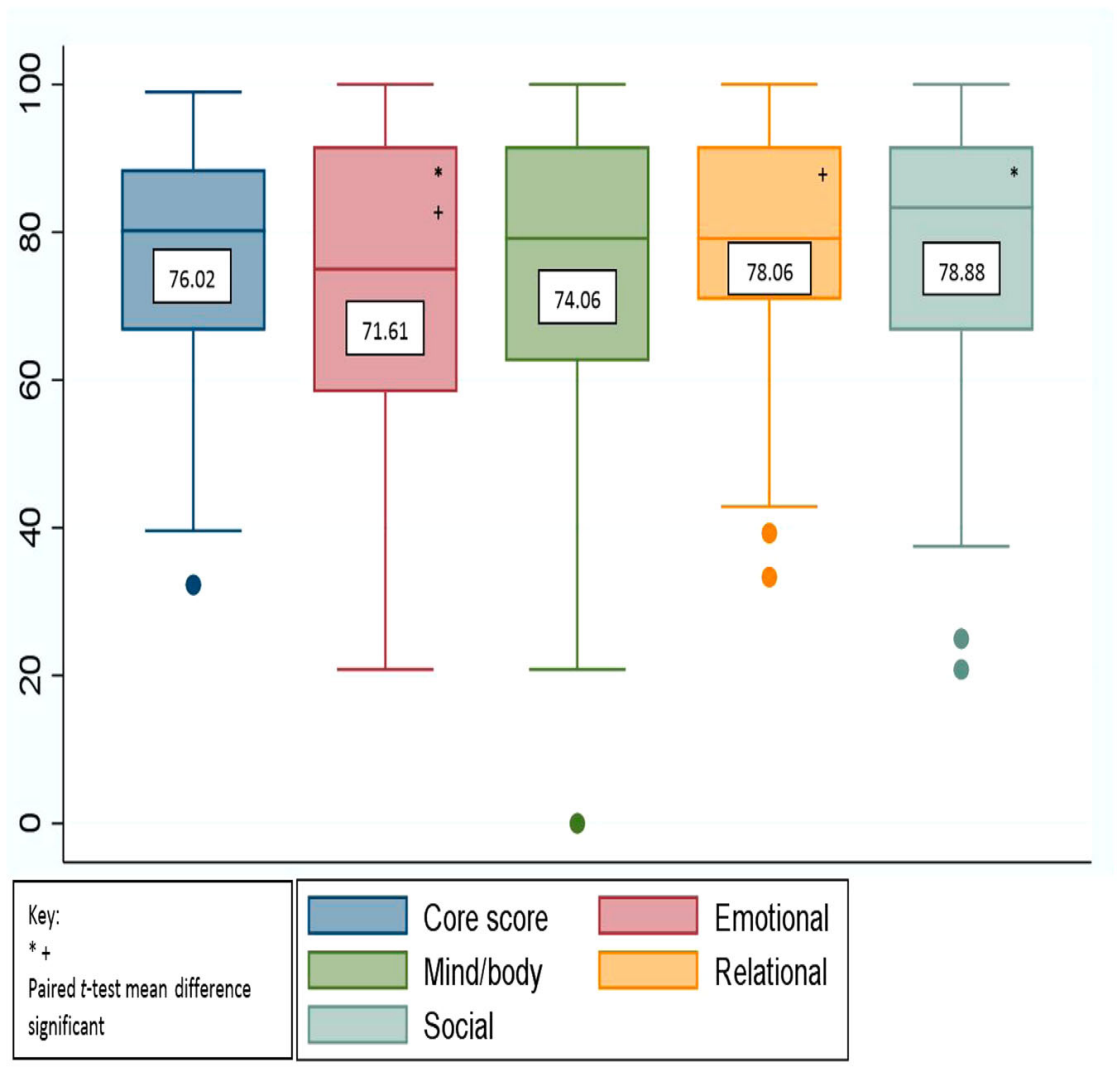
Distribution and Mean FertiQoL core and domain scores for the sample, *n* = 102. This box and whisker plot shows the distribution of the FertiQoL core and domain scores in this sample. In this figure, minimum (lower whisker), first quartile (bottom of the box), median (line in the middle of the box), third quartile (top of the box) and maximum (upper whisker) are displayed. Means are written in the centre of each box. The dots indicate outliers in the sample. The * and + indicate that paired t-tests of means for those scores were statistically significant at *p* < 0.05.

The mean core FertiQoL score for the sample was 76.02 (SD = 16.26), and mean scores for the domains were: emotional 71.61 (SD = 22.04), relational 78.06 (SD = 16.62), mind/body 74.06 (SD = 22.53) and social 78.88 (SD = 18.24), see Figure 2.

The mean score for the emotional domain (*M* = 71.61, SD = 22.04) was significantly lower than the social domain (*M* = 78.88, SD = 18.24); *t*(202) = 2.57, *p* = 0.01 and the relational domain (*M* = 78.06, SD = 16.62); *t*(202) = 2.36, *p* = 0.01. All other domains did not differ significantly from each other.

#### Factors associated with the FertiQoL

**Figure 3.**
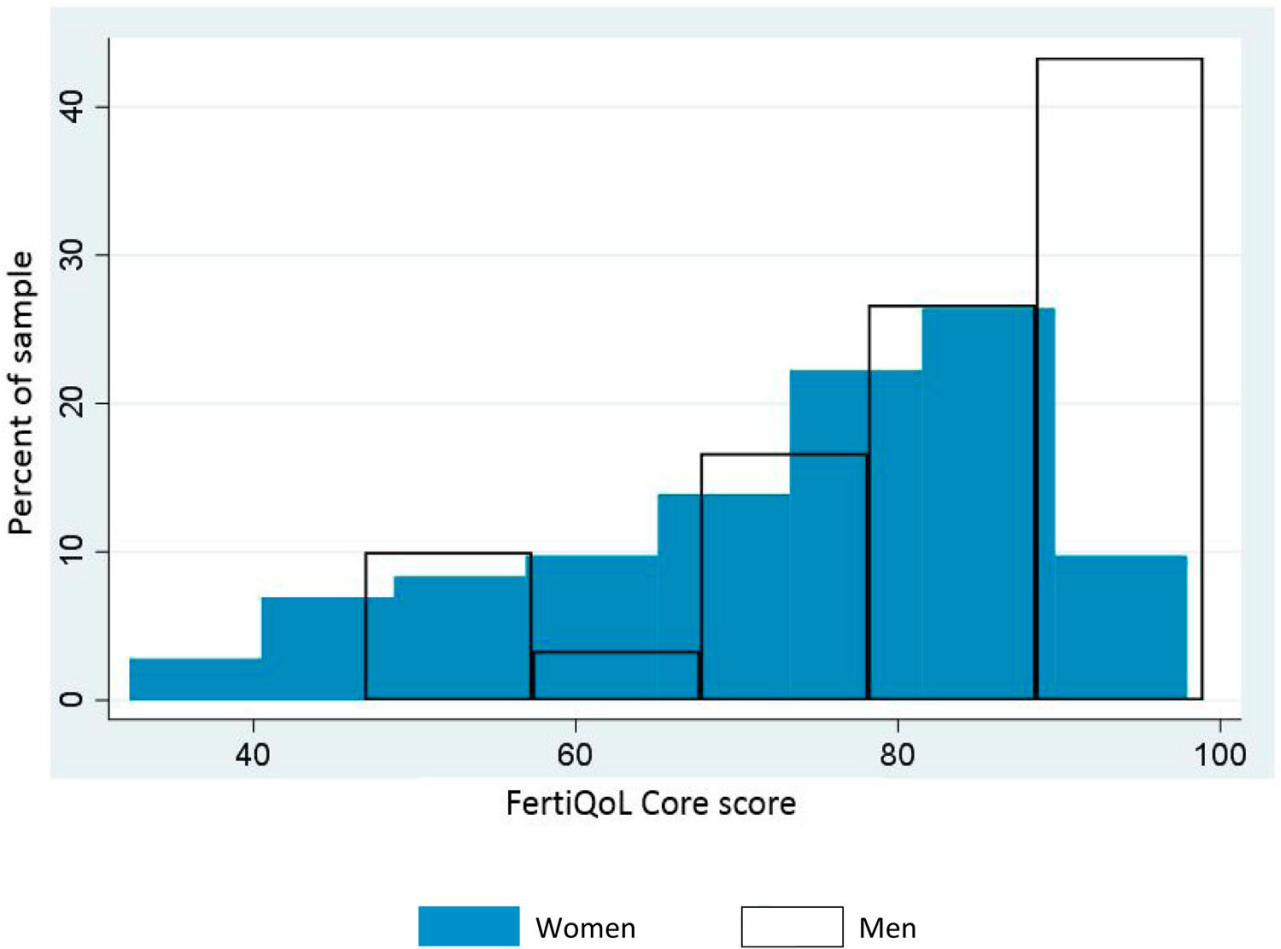
Distribution of FertiQoL core score by GENDER. This figure shows the difference in the distribution of FertiQoL core scores for men and women. The blue distribution is for women’s scores and the overlay outline distribution is for men’s scores. The figure shows that both distributions were skewed to the right (higher scores), however, most men scored much higher than women, and women had a wider distribution of scores. This indicated that in this sample, while both men and women reported high scores, on average men scored higher than women and there was more variability in the women’s scores.

Table 2 shows the results of the regression model. We examined the relationship between FertiQoL and the health and socio-demographic variables and the only factor with a significant association with the FertiQoL core score was gender. Women scored nine points lower than men on the FertiQoL core score (*β* = −9.05, CI = −16.51 to −1.59, *p* < .05), see Figure 3 for distribution of core score by gender. Women scored 8 points lower than men on the FertiQoL relational score (*β* = −8.55, CI = −16.33 to −0.76, *p* < 0.05) and almost 14 points lower on the FertiQoL emotional score (*β* = −13.88, CI = −23.80 to −3.96, *p* < 0.01).

**Table 2.** Regression model demonstrating the relationship between FertiQoL core and domain scores with the health and socio-demographic variables.

Variables	*Beta* coefficient (unstandardized) for FertiQoL Scores (*n* = 102)				
	Core score	Emotional domain	Relational domain	Social domain	Mind/body domain
Age > 35 years	4.02	7.35	−0.65	4.07	3.23
Woman	−9.05**	−13.88**	−8.55**	−6.17	−9.32
Urban	−1.07	−1.49	3.13	−3.75	−0.80
Education > Secondary school	5.68*	6.97	2.40	4.77	7.84*
Duration of infertility > 1 year	1.16	2.15	4.52	0.32	−3.07
Infertility (female factor only)	−4.68	−7.17	2.17	−5.92	−7.73*
Consanguineous marriage	3.20	3.32	4.23	0.89	4.59
Mean	76.02	71.61	78.06	78.88	74.06

Note: FertiQoL = Fertility Quality of Life Tool; *p < 0.10; **p < 0.05.

### Objective 2: core themes from in-depth interviews

Four themes emerged from the thematic analysis of the interview data: (a) ‘sense of something missing’; (b) ‘spousal bond continuum’; (c) ‘sense of social pressure’ and (d) ‘coping strategies’, see Table 3 for themes, sample sub-themes, codes and quotes.

**Table 3.** Analytic scheme for themes that emerged from qualitative data analysis displaying the four themes, sample sub-themes, codes and quotes.

Analytic scheme			
Theme	Sample sub-theme	Code	Quote
1. Sense of ‘something missing’	Something missing	Emotional – something is missing – incomplete family	‘it has affected me from the emotional side, one doesn’t feel at ease emotionally, as if something is missing, yes settled, working, the whole family is well, socially OK, everything is excellent but one feels something is missing.’
2. Spousal bond continuum	Unmet need for spousal bond	Relational – desire to have a child – make husband feel a ‘man’ Relational – desire to have a child – to please husband	‘I want to have a baby, I want to be settled, I want my husband to feel he is a man, with a home, family, kids.’ ‘I want to do this thing for my husband to make him happy with this thing. Not because I have a strong desire to get pregnant, especially at this time’
3. Social pressure	Pressure to conform to social norms	Social – expectation – have children	‘in-laws, family, ‘you still haven’t become pregnant? You’re not three yet? Don’t you want to go to the doctors? You’re not concerned about this issue?’’
4. Coping strategies	Faith based coping	Coping – Faith – Surrendering to God’s will	‘I feel like why haven’t I had [a child], but this thing is from God (God’s will), all of it.’

#### ‘Sense of something missing’

The internal emotional impact of infertility was a pervasive theme, many of the participants noted that the lack of biological offspring left them with a feeling of ‘something missing’. For example, when asked how infertility had affected her, a 42 year-old woman:

It has affected me from the emotional side, one doesn’t feel at ease emotionally, as if something is missing, yes settled, working, the whole family is well, socially OK, everything is excellent but one feels something is missing. [She further explained the idea that not having a biological child makes life incomplete, she said] … one feels that one marries to form a family … when this thing is missing, there is a lack, and life is not complete. (PT 7)

#### ‘Spousal bond continuum’

We observed reports about the impact of infertility on the spousal relationship with varying degrees of spousal support. Some participants reported supportive spouses who understood what they were feeling. For example, a 35-year-old woman:

No, no there is no effect on my relationship with my husband thank God. He tells me ‘everything has its time, if God wills it, it [the child] will come’. And I was thinking that maybe he was thinking about something [another wife], or he was upset, but thank God no. (PT 4)

Several women reported negative interactions with their husbands as a result of infertility. For example, a 28-year-old woman:

I just started to fight with my husband … I’ve just been telling him that he has to come [home to try to conceive again], and if he doesn’t come then maybe we can separate, time is passing, and time is affecting me not him. (PT 10)

In addition, consequences like polygamy were reported, a 22-year-old woman:

He will marry [again] because that’s what happens, he said ‘if you don’t have kids after some time, I will get married’! (PT 12)

There were also women who wanted to have a child to please their husbands. For example, a 35-year-old woman:

… I want to have a baby, I want to be settled, I want my husband to feel he is a man! With a home, family, kids. (PT 1)

#### ‘A sense of social pressure’

Participants reported feeling pressure from questions from family, friends and society in general. Many of the participants reported that shortly after marriage people in their social context started asking them about becoming pregnant, ‘haven’t you become three yet?’ A woman, aged 43, stated that these questions can make you evaluate your situation differently:

Yes, they make you aware that you have a problem, they talk too much … especially the aunts and grandmothers they are the ones nagging the most … but if you are alone and you think about it, you wouldn’t assess it this way. (PT 14)

#### ‘Coping strategies’

We observed different coping strategies that participants employed to deal with their infertility, that can be categorized as faith-based and non-faith-based coping, see Table 4. An example of faith-based coping came from a 29-year-old woman:

I have the desire for a child, but what can you say, ‘gismat rab al a’alameen’ [Arabic for God’s will]. (PT 19)

**Table 4.** Illustrative quotes demonstrating when faith-based coping and non-faith-based coping were used in relation to the other three main themes.

Type of coping (description)	Themes		
	Social pressure	Internal distress (Sense of ‘something missing’)	Spousal (Spousal bond continuum)
Faith based coping: Using faith as a way to accept one’s situation	1: in-laws, family, ‘you still haven’t become pregnant? You’re not 3 yet? Don’t you want to go to the doctors? You’re not concerned about this issue?’ inside you, you wish you could become a mother, there isn’t a woman who doesn’t wish to become a mother. But I have surrendered to the fact that this thing is within God’s hands, when it comes it comes. What God wants will come, so the worry … everything is coming from God.	4: walahi, maybe for a while I have been emotionally uncomfortable/uneasy. But in the end this is God’s will, I used to get nervous (uneasy) after the miscarriages, because I used to get pregnant and miscarry and I didn’t know the reason, and the tests were clean, I used to get nervous, but after I had the baby (still born after 9 months’ gestation) it was fine. Alhamdulillah (thank God), God willed it so (that the baby would die in-utero), my reaction was a’adee (normal).	RRB: Ok so this thing upsets you, that he could marry again or something? 12: maybe, of course I get upset RRB: what do you feel? 12: I feel like there is something lacking in me (less than or inferior) to begin with, I feel like why haven’t I had, but this thing is from God (God’s will), all of it.
Non-Faith based coping: Using non-faith coping mechanisms like meaning making or behavioural change to accept one’s situation	15: yes, I want a baby, but I think it’s early, I just got married.	13: Bit by bit, it gets more a’adee (acceptable, bearable), but in the first months, every time it came (period), I would be in a state of worry and disturbance that pregnancy hadn’t occurred. 5: I feel I can still have a baby, there is lots of hope. 16: I look at myself (situation) and see that I’m not mustagira (not residing together), if we lived together of course there would be (kids). 17: I feel like I haven’t been going (to treatment) yet, I have just started (being at the clinic), so I’ve not lost hope yet, going well so far.	11: of course, they ask him, but he doesn’t show (tell) me at all. And of course, this is not something that needs (explanation), he communicates it to me with humour so as not to hurt my feelings.

Note: FQ = FertiQoL; RRB = Interviewer.

An example of non-faith-based coping came from a 30-year-old woman who is not living with her husband:

I look at myself [situation] and see that I’m not ‘mustagira’ [Arabic for not living together], if we lived together of course there would be [kids]. (PT 16)

#### Interconnectedness of themes

**Figure 4.**
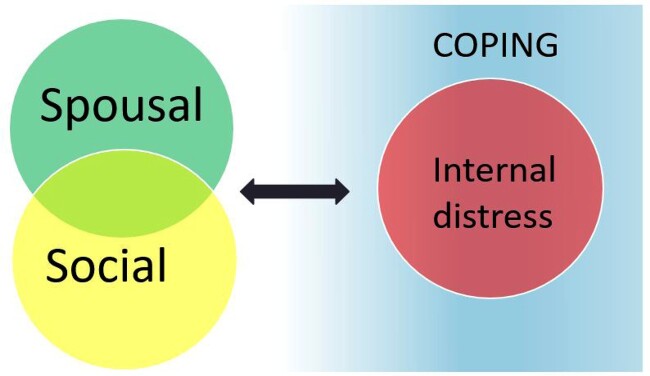
Conceptual framework for qualitative analysis, the thematic map of interconnectedness of themes. In this figure, the thematic map illustrates the interconnectedness of the four main themes that emerged through thematic analysis. The figure shows that the impact of infertility on social or spousal relationships would require coping if it led to a state of internal distress. The figure also shows that spousal and social themes overlapped.

Further analysis indicated that the core themes were not independent, leading to the development of a conceptual framework, thematic map of interconnectedness of themes, see Figure 4. The thematic map showed that the impact of infertility on social or spousal relationships would require coping if it led to a state of internal distress. For example, the following quote from a 35-year-old woman, illustrates how the social impact and pressure from the questions lead to negative feelings which in turn required coping:

… yes, it has affected me, people’s questions, even my immediate family (parents, siblings) have told me to try to seek medical help abroad ‘go get this checked out, maybe there is something, why haven’t you had a baby? why haven’t you become pregnant?’, but I’m now convinced that everything is from God. (PT 4)

### Objective 3: triangulation of the data

In general, it appeared that the first three themes were more common while the coping theme was less frequent and largely related to internal distress. It could be that participants were focused on distress because they had been asked to think about their problem in the IDIs. Non-faith-based coping was more common than faith-based. This is surprising given that Sudanese culture emphasizes the dependence on religious doctrine to understand and cope with hardship. When comparing themes with quantitative data, it appeared that those who reported faith-based coping were younger, had been infertile for a shorter duration, were more likely to have had a previous pregnancy and less likely to have female factor infertility only.

#### Gender differences

Since gender (being a woman) was the only factor found to be significantly associated with FertiQoL scores in the quantitative analysis, we conducted further examination to triangulate the data by exploring the qualitative themes separated by gender, see Table 5. We noted the following differences: (1) two of the three men denied any impact on their QoL; and (2) women were able to elaborate the emotional and relational aspects of the experience, while none of the men did. These findings may help explain why men scored higher overall and on emotional and relational domains but not why their mind/body and social domain scores were comparable. This led to further examination of those domains. None of the participants mentioned impact on the mind/body domain in Group B (IDIs). While none of the men elaborated on social impact, almost all the women (12 of 17, 70.6%) did.

**Table 5.** Triangulation of thematic analysis of coping by gender, using illustrative quotes to help explain results of the regression analysis that identified gender as an independent factor associated with FertiQoL core, emotional and relational domain scores.

Triangulation of thematic analysis of coping by gender^a^			
Themes unique to each gender	Quotes from men	Quotes from women	Potential explanation for why and how Gender was associated with FQ scores
Denial of impact of infertility	RRB: I want you to tell me how you feel this issue has affected your life, e.g. emotionally, with each other, socially etc. 18: walahi for me I don’t feel there is an effect, ya’anee (sort of) no effect, nothing, yeah RRB: not in any aspect of your life? 18: nothing at all When asked about how infertility has affected his life PT 8 answered one question and shrugged at all attempts to elaborate further. RRB: has your fertility problem affected your life? 8: yes it has an effect, but not 100% effect	None	This denial of problems by men can help explain why men scored higher in the core score, because they would have denied an impact in the FQ as they did in the interview. This appears to be independent of whether there was an impact or not. Meaning that regardless of whether you ask in interview open ended questions or FQ there is less reporting of impact (which could be underreporting or just that it has less impact).
Themes common to both genders	Quotes from men	Quotes from women	
1. Emotional (Sense of something missing)	2: it’s been 5 years and God has not given me a child, and this is what’s affecting me, a lot for me, it’s really hard for me … you have kids, you raise them, you educate them so that when they grow up they can carry you (take care of you) … it makes me feel upset and unable to concentrate	12: walahi, yes it upsets me, but not emotional problems or something like that, thank God. I mean I haven’t had a baby … why … something like that. I’ve been searching for treatment for how long now, I’m just imploring God. 13: So, the emotional effect is always huge. I can’t tell you, with every period you have this emotional reaction (infee3al, like an outburst) and zahaj (literally translates to boredom but is used to describe being emotionally upset) and disturbance (meaning emotional disturbance), just this awful thing (state). 7: you mean not having a baby? Walahi, it has affected me from the emotional side, one doesn’t feel at ease emotionally, as if something is missing, yes settled, working, the whole family is well, socially OK, everything is excellent but one feels something is missing.	Only one of the three men reported an emotional impact, while almost all women did (14 of 17, 82.4%) which can help explain why women scored lower on the FQ emotional domain
2. Spousal bond continuum	RRB: which aspect do you feel has been affected the most? 2: with my wife and society (and he didn’t elaborate despite further probing)	RRB: so how has it affected your relationship with your husband? 1: No, not at all, at some point there was pressure that ‘this woman has not had a child’, ‘you should get married’. 13: walahi (oath), I desire a baby, of course, I wish for this thing, and mostly to make my husband happy, because he likes this thing more, but I like kids, but not to that extent (as much as her husband). I feel the disappointment because I want to do this thing for my husband to make him happy with this thing. Not because I have a strong desire to get pregnant, especially at this time	Only one of the three men reported a spousal impact, while almost all women (15 of 17, 88.2%) reported some spousal impact, which can help explain why women scored lower on the FQ relational domain
3. Social pressure	RRB: which aspect do you feel has been affected the most? 2: with my wife and society (and he didn’t elaborate despite further probing)	20: walahi, with my husband I have no problem, but with social relationships, whenever I meet someone they always ask ‘aha not three yet? What’s happening? What have you done so far (regarding treatment)?’ they always ask questions. 4: yes, it has affected me, people’s questions, even my immediate family (parents, siblings) have told me to try to seek medical help abroad ‘go get this checked out, maybe there is something (that can be done)’ In the clarification of the social isolation question of the FQ: RRB: what I mean is that do you avoid social situations like weddings and such? 9: I just go straight away Then for the social pressure question of the FQ: 9: yes, they talk, but not my family RRB: people outside the family? 9: yes RRB: does this thing upset you? 9: yes, but not a lot Then after I showed her the results of the FQ: RRB: ok on the social side 91%, so not much impact at all. What do you think about that? 9: shrugs RRB: I mean do you agree there is not much impact in this area? 9: yes RRB: so it’s not having an impact, in spite of the comments, you’re continuing with your life as always? 9: yes, continuing as always RRB: but what they say bothers you? 9: yes	Only one of the three men reported a social impact, while almost all women (12 of 17, 70.6%) reported some social impact. However, it appeared that the codes all related to being asked too many questions and the pressure this creates, while the FQ social domain questions mainly pertain to social engagement. It would appear that in Sudan, where social engagements are perceived as obligations, regardless of what impact the social pressure might have this doesn’t change the level of engagement and this can help explain why women and men’s score on the FQ social domain didn’t differ as can be seen from the interview with PT 9.
4. Coping strategies	2: it’s been 5 years and God has not given me a child, and this is what’s affecting me, a lot for me, it’s really hard for me RRB: when you look at these results, how does it make you feel? 8: a really wonderful feeling	1: inside you, you wish you could become a mother, there isn’t a women who doesn’t wish to become a mother. But I have surrendered to the fact that this thing is within God’s hands, when it comes it comes. What God wants will come, so the worry … everything is coming from God. 16: I look at myself (situation) and see that I’m not mustagira (not residing together), if we lived together of course there would be (kids).	It appears that both men and women used faith based and non-faith based coping.

Note: FQ = FertiQoL; RRB = Interviewer.

^a^ Regression analysis identified Gender as an independent factor associated with FertiQoL core scores, emotional and relational domain scores, meaning women and men scored significantly different on these three scores. This table provides illustrative quotes to help explain the difference between men and women in this sample.

## Discussion

### Principal findings

Results indicated that infertility negatively impacted quality of life of Sudanese patients, especially the emotional aspect, as measured quantitatively by the FertiQoL. Average core score for the current sample was higher than average core score in the published literature (Koert et al., 2019), but within the range of core scores in those countries. For example, the mean core score in this sample was 76 which is comparable to Jordanian samples, which ranged from 64.2 to 71.3 and Turkish samples from 65.2 to 76.5 (Koert et al., 2019). The higher mean core score might have been impacted by factors such as psychological vulnerability, gender and culture, previously found to be associated with fertility-related QoL (Koert et al., 2019). Gender was the only variable associated with FertiQoL core scores, consistent with findings in other countries that women consistently reported lower fertility-related QoL than men (Koert et al., 2019). In this sample, women reported more impact on their quality of life overall and specifically the emotional and relational aspects. Results indicated that men were reluctant reporters across all methods used, congruent with the literature (Call & Shafer, 2018; Martin, Neighbors, & Griffith, 2013; Smith, Mouzon, & Elliott, 2018). Results of this study indicated that quantitative and qualitative methods can be complementary, in this instance confirmatory.

The pervasive theme of ‘sense of something missing’ as a result of childlessness, may be related to the fact that in Sudan children are viewed as the ultimate goal of marriage and that in Islam offspring are valued as gifts from God akin to wealth (Quran, Surah Al-Kahf [18:46]). The sense of lack or incompleteness is not unique to Sudan since parenthood has been identified as a central human life goal (Daniluk, 2001; Gameiro & Finnigan, 2017) and childlessness can be a ‘blocked goal’ that negatively impacts wellbeing, even in western/high income countries (Da Silva, Boivin, & Gameiro, 2016; Hansen, Slagsvold, & Moum, 2009).

Given that martial dysfunction, divorce and polygamy have been reported as consequences of infertility (Rouchou, 2013), we anticipated that infertility would impact the spousal relationship negatively. While that was true for some women, others reported supportive and understanding husbands, similar to some published reports of Arab men (Inhorn, 2012). Some women reported wanting to have children to ‘please’ their husbands or preserve their manhood. Those women might have felt their infertility cast doubt about their husbands’ sexual ability, perpetuated by the myth of fertility being an indicator of the man’s sexual virility commonly held in Sudan (Al Safi, 2007) and other LMIC (Inhorn, 2012; Widge, 2002).

Triangulation added to the understanding of coping with infertility. It appeared that participants were focused on describing their problem rather than coping. Surprisingly there was less mention of faith-based coping than one would expect in a Muslim country. Participants might have been less religious as a group or faith-based coping might have been presumed as a ‘given’, so they only report coping beyond that. Given that faith-based coping was reported by younger participants and those who had shorter durations of infertility, it could be that when the infertility problem has just started or one is younger (has more time), then the default coping is faith-based (first resort), and more ‘active’ non-faith based coping was utilized when the problem was more complex or severe or longer. It could also be that it is a default that is not elaborated. Reports in the literature suggest that as infertile individuals get older and the likelihood of having biological offspring is reduced, they require more support to cope with the finality of their situation (Gameiro & Finnigan, 2017; Hansen et al., 2009). Moreover, the longer duration of infertility compromises the effectiveness of coping (Zurlo et al., 2018), requiring different or additional coping strategies.

Triangulating the data also lead to potential explanations of the gender differences noted (men had significantly higher FertiQoL core scores). Men’s higher scores could have been because they denied any potential impact in the FertiQoL. There are several possible explanations for men’s inability or reluctance to discuss topics, including emotional and spousal issues as compared to women who were more engaged and expressive in the interviews. First, the differences might reflect a real lack of impact felt by men, though this would be inconsistent with a growing body of research demonstrating that men are negatively impacted by infertility (Fisher & Hammarberg, 2012). Second, women, in general, are more verbally and emotionally expressive (Hyde & Linn, 1988; Wester, Vogel, Pressly, & Heesacker, 2002). Third, it is well documented that men tend to underreport internalizing symptoms like sadness (Call & Shafer, 2018; Smith et al., 2018), consequently, internalizing problems like depression can be underdiagnosed using standard measures (Martin et al., 2013). Fourth, it could be that women were more elaborative because of how they processed this topic. According to the Elaboration likelihood model (ELM) (Cacioppo & Petty, 1984) topics processed centrally can be elaborated, compared to peripheral processing, because central processing requires increased motivation and ability related to the topic. In the case of infertility, women are more motivated to become mothers for biological or social reasons and/or to avoid the harsh consequences such as polygamy or divorce. Additionally, women’s familiarity with their reproductive processes such as menstruation and how those relate to childbearing can increase their perceived ‘ability’ in this area. In addition to ‘global’ explanations, a localized explanation of why Arab men may deny or be hesitant to talk about infertility could be related to pervasive myths equating sexual virility with fertility (Al Safi, 2007) leading to hesitance in assuming responsibility for infertility (Inhorn, 2012). Society has also provided men with polygamy or divorce as socially sanctioned solutions for childless marriage (Inhorn, 2012). This potentially reduces the impact of infertility on the men, unlike their wives, who bear the responsibility and have limited options (Al Safi, 2007; Inhorn, 2012).

Although there were no major differences for when faith-based coping versus non-faith-based coping were used, both types of coping were necessary when the lived experience (e.g. spousal tension/social pressure) lead to internal distress, congruent with the ‘Stress appraisal and coping’ model (Lazarus & Folkman, 1984). According to this model, a stressor only requires coping if it is appraised to be harmful, dangerous or challenging and exceeds the available resources. This model was adapted for infertility with the addition of social support and personality factors (Gourounti, Anagnostopoulos, & Vaslamatzis, 2010). The current findings indicated that social impact is multifaceted and can be a hindrance or support in appraising and coping with infertility, therefore, the Gourounti et al. (2010) model can be further adapted to widen social support to be more inclusive of other social issues, especially social pressure.

### Strengths and limitations

The main limitation was the use of a convenient sample that was homogeneous, mostly educated women attending a fertility clinic. Since all the participants were in treatment, it cannot be assumed that they are representative of infertile individuals in the community who did not seek treatment. The small sample size limited generalizability of the results, however, generalizability was not the purpose of this study since the FertiQoL has been validated in numerous studies with large samples (Koert et al., 2019). Instead, the purpose of the current study was to use qualitative and mixed methods to understand peoples’ perspectives and personal stories and to compare cases to discover patterns (Patton, 2014), therefore, the small sample size had less impact on results (Cleary, Horsfall, & Hayter, 2014). Finally, there might have been caveats about data collection methods such as impact of a woman interviewer (gender discordant for men participants), which have been shown to impact interviewees in some studies (Catania, Binson, Canchola, Pollack, & Hauck, 1996; Huddy et al., 1997; Kane & Macaulay, 1993). The impact of the woman interviewer is unclear because during the interviews while discussing issues not related to the quality of life, such as past medical history, the men were as forthcoming as the women.

The main strength of the study was the use of a mixed-methods approach, known to enhance the rigour of the study through triangulation and providing explanatory factors necessary to understand/support the quantitative data (Creswell & Clark, 2009). We used a mixed-methods approach to understand the underlying issues that might contribute to the quantitative data in the current study, and published studies, since the FertiQoL has not been studied qualitatively. Triangulation of data increased the validity of study findings and revealed aspects that might have otherwise been overlooked (Carter, Bryant-Lukosius, DiCenso, Blythe, & Neville, 2014). Adherence to best practices guidelines of qualitative analysis (Braun & Clarke, 2006; Critical Appraisal Skills Programme, 2014; Meyrick, 2006) strengthened the study. The research team’s multinational and multidisciplinary nature meant extensive familiarity with the subject, the culture and the methodology, leading to enhanced trustworthiness of the results.

### Implications

Findings have implications for interventions for infertile individuals. Fertility related QoL appears to be more closely related to cognitive appraisal and support than to health and social determinants, congruent with previous research (Gourounti et al., 2010). Therefore, treatment should help infertile individuals appraise their childlessness differently, encourage them to seek social support and inform them about when to seek psychosocial intervention. If childlessness becomes a more permanent state then research shows that disengaging from that ‘blocked goal’ and engaging with alternate life goals can improve wellbeing (Da Silva et al., 2016). Reproductive healthcare professionals should be trained to provide support and to refer complex cases to the appropriate specialists. In Sudan and regionally, systemic vulnerabilities regarding the availability, accessibility, affordability, and quality of psychosocial support should be addressed in research and policy.

Results indicated that FertiQoL scores, corresponded to the subjective experience reported in the interviews, enhancing the credibility of the FertiQoL in this population. This is important because of the increased interest and uptake in the utilization and validation of the FertiQoL in socially diverse settings as indicated by the number of studies and translations in non-western countries (Asazawa & Mori, 2015; Chi, Park, Sun, Kim, & Lee, 2016; Goker et al., 2018; Li, Long, Liu, He, & Li, 2016; Maroufizadeh et al., 2017; Valsangkar, Bele, Bodhare, & Sai, 2011) and non-English speaking European countries (Cserepes et al., 2014; Järvholm, Johannesson, Clarke, & Brännström, 2015).

### Future research

Since this was a small, homogenous sample, determining external validity and generalizability requires replication with bigger and more diverse samples within different contexts. In the current study, as was the case for most research on the FertiQoL, the sample was mostly women in clinical settings. Therefore, future research should include men and non-clinical samples. Men’s fertility-related QoL should be examined using different innovative methodologies to capture the true nature of the impact (e.g. IDIs with men interviewers, focus groups, anonymous online surveys). These methods could potentially overcome difference in ability to express and elaborate on emotional issues. Qualitative and quantitative studies should be replicated with infertile individuals not in treatment, in community-based studies. Qualitative research should be replicated with culturally diverse groups, to determine whether the themes that emerged are common across cultures. Insights about how to interpret scores in different cross-cultural contexts should also be considered.

## Conclusion

Results of this study indicated that infertility negatively impacted the QoL of these Sudanese individuals, and women were worse off. Current results combined with established knowledge about fertility-related QoL indicated that cognitive appraisal, social support and social pressure were more impactful on fertility QoL than health and social determinants. Therefore, infertile individuals should be encouraged to enhance their coping by seeking support that can help them appraise their infertility differently and manage social pressure. Results supported the utility of FertiQoL to assess QoL in LMIC like Sudan and highlighted the need for further qualitative examination of the cross-cultural applicability and interpretation of the FertiQoL in diverse populations.

## Supplementary Material

Supplemental Material

## Data Availability

The datasets used and/or analysed during the current study are available from the corresponding author on reasonable request.
