# Supplementary material for: Quality of life of Sudanese patients attending a fertility clinic: a mixed methods study
Source: Health Psychol Behav Med. 2021 Dec 1;9(1):1006–30. doi: 10.1080/21642850.2021.2007773 (PMC8648023; doi:10.1080/21642850.2021.2007773)
Supplement: Supplemental Material [file RHPB_A_2007773_SM0028.docx]

**Supplemental Materials**

**Procedure for survey and interviews:**

- Recruit study participants from clinic waiting area
- Take participants to private room
- Explain the study and provide the consent form

1. **Survey:**
2. Collect the signed consent form
3. Provide the printed survey
   1. Background information form
   2. Arabic FertiQoL
4. Collect the survey
5. Check all items are completed
6. Debrief and thank the participant
7. **Interview:**
8. Explain audio recording of interview
9. Collect the signed consent form
10. Obtain answers to the background information form
11. Use topic guide for interview
    1. Ask questions as in the topic guide
    2. Administer FertiQoL verbally
    3. Use online FertiQoL scoring
    4. Show the participants their FertiQoL scores and discuss those
12. Debrief and thank the participant

**List of materials:**

1. Arabic consent form for the survey
2. Arabic consent form for the interview
3. English Background Information Form
4. Arabic Background Information Form
5. Arabic FertiQoL
6. English and Arabic FertiQoL interview topic guide
7. Arabic debrief

**Arabic Consent form for the survey**

**أستمارة الموافقة في استبيان عن FertiQoL**

أنا أفهم أن مشاركتي في هذا المشروع تتضمن تقديم أفكاري ومشاعري حول FertiQoL أثناء اداء الاستبيانات واستبيان عن معلوماتي الديموغرافية (على سبيل المثال، العمر، والتعليم). ستستغرق الدراسة حوالي 10 إلى 15 دقيقة .

وأنا أفهم أن مشاركتي في هذه الدراسة طوعية تماما وأستطيع الانسحاب من الدراسة في أي وقت دون إبداء أسباب وأنا حر أن أسأل أي سؤال في أي وقت. أنا حر في الانسحاب أو مناقشة مخاوفي مع الباحثة رشا بيومي.

أنا أفهم أن سيتم تخزين المعلومات التي قدمتها على جهاز كمبيوتر (محمي بكلمة مرور) المملوك لدى الباحثة رشا بيومي والأستاذة جاكي بوافين, وسوف يحفظ من غير اسم ، بحيث أنه من المستحيل أن تتبع هذه المعلومات لي على حدة. و سيتم الاحتفاظ بالبيانات والردود على الاستبيان إلى أجل غير مسمى.

أفهم أيضا أنه في نهاية الدراسة ستقدم لي معلومات حول الغرض من الدراسة.

أنا، ___________________________________ (الاسم) موافق على المشاركة في الدراسة التي سوف

تجريها رشا بيومي (كلية علم النفس، جامعة كارديف مع إشراف البروفيسور جاكي بوافين).

التوقيع: ___________________________________

التاريخ: ___________________________________

**Arabic Consent form for the interview**

**أستمارة الموافقة في استبيان عن** FertiQoL

أنا أفهم أن مشاركتي في هذا المشروع تتضمن تقديم أفكاري ومشاعري حول FertiQoL أثناء اداء الاستبيانات. سيتم بعد ذلك إكمال مقابلة قصيرة واستبيان عن معلوماتي الديموغرافية (على سبيل المثال، العمر، والتعليم). ستستغرق الدراسة حوالي 10 إلى 20 دقيقة .

وأنا أفهم أن مشاركتي في هذه الدراسة طوعية تماما وأستطيع الانسحاب من الدراسة في أي وقت دون إبداء أسباب وأنا حر أن أسأل أي سؤال في أي وقت. أنا حر في الانسحاب أو مناقشة مخاوفي مع الباحثة رشا بيومي.

أنا أفهم أن المقابلة ستكون مسجلة حتى يمكن نسخها ودراستها. سيتم تخزين المعلومات التي قدمتها على جهاز كمبيوتر (محمي بكلمة مرور) المملوك لدى الباحثة رشا بيومي والأستاذة جاكي بوافين, وسوف يحفظ من غير اسم ، بحيث أنه من المستحيل أن تتبع هذه المعلومات لي على حدة. وبمجرد أن يتم نسخ تسجيل المقابلة سيتم مسحه و الاحتفاظ بالبيانات والردود على الاستبيان إلى أجل غير مسمى.

أفهم أيضا أنه في نهاية الدراسة ستقدم لي معلومات حول الغرض من الدراسة.

أنا، ___________________________________ (الاسم) موافق على المشاركة في الدراسة التي سوف

تجريها رشا بيومي (كلية علم النفس، جامعة كارديف مع إشراف البروفيسور جاكي بوافين).

التوقيع: ___________________________________

التاريخ: ___________________________________

**English Background Information Form**

1. Patient number ______________
2. Age ______________
3. Sex ______________
4. Address ______________
5. Occupation ______________
6. Education:
   - Illiterate:
   - Primary level:
   - Secondary level:
   - More than secondary level:
7. Duration of marriage ______________
8. Duration of couple living together ______________
9. Are you married to a relative?
10. Have you undergone female circumcision?
11. Menstruation:
    - Normal
    - Not normal
12. If menstruation is not normal:
    - No period > 6 months
    - No period < 6 months
    - Increase in the amount of menstruation
    - Decrease in the amount of menstruation
13. Painful intercourse: Yes/No
14. Medical and surgical history:
    - Blood pressure (hypertension)
    - Thyroid disease
15. Have you been pregnant before: Yes/No
16. Number of previous pregnancies ______________
    - Without treatment ______________
    - With ovarian stimulation only ______________
    - With ART ______________
17. Duration of delay in pregnancy ______________
18. Reasons for delay in pregnancy:
    - Husband
    - Wife
    - Both
    - Unknown

**Arabic Background information form**

معلومات عامة وطبية

1. الرقم ______________

2. العمر ______________

3. الجنس ______________

4. العنوان ______________

5. المهنة ______________

6. التعليم: أمي___ المرحلة الابتدائية___ المرحلة الثانوية___ أكثر من مستوى ثانوي__

7- مدة الزواج ______________

8- مدة إقامة الزوجين معا ______________

9. هل أنت متزوج من أقاربك: نعم_____ لا_____

10. هل خضعتي لختان الإناث: نعم_____ لا_____

11. الدورة الشهرية: طبيعية_____ غير طبيعية_____

12. إذا كانت الدورة الشهرية غير طبيعية:

• غياب أكثر من 6 أشهر

• غياب أقل من 6 أشهر

• زيادة في كمية الحيض

• انخفاض في كمية الحيض

13. ألم حادأثناءالعلاقة الزوجية: نعم_____ لا_____

14. التاريخ الطبي والجراحي:

• ضغط الدم (ارتفاع ضغط الدم)

• مرض الغدة الدرقية

• داء السكري

• فقر الدم المنجلي

• السرطان

• مرض الكلى

• مرض السل

15. هل حدث لك حمل من قبل: نعم_____ لا_____

16- عدد مرات الحمل السابق ______________

• بدون علاج ______________

• بعد العلاج بالمنشطات فقط______________

• بعد العلاج بتقنيات مساعدة للخصوبة______________

17 - مدة تأخر الحمل ______________

18- أسباب تأخرالإنجاب:

• الزوج

• الزوجة

• الزوجين معا

• لا يوجد سبب واضح

**ﻓﻴﺮﺗﻜﻮل اﻟﺪوﻟﻴﺔ**

**FertiQol International**

# اﺳﺘﺒﻴﺎن اﻟﺨﺼﻮﺑﺔ و ﻧﻮﻋﻴﺔ اﻟﺤﻴﺎة ﻟﻌﺎم (2008) ﻟﻜﻞ ﺳﺆال، ﻟﻄﻔﺎ ﺗﺄآﺪ )ﺑﻮﺿﻊ ﻋﻼﻣﺔ ﻓﻲ اﻟﻤﺮﺑﻊ( ﻣﻦ أن اﻹﺟﺎﺑﺔ ﺗﻌﻜﺲ أآﺜﺮ و ﻋﻦ آﺜﺐ آﻴﻒ ﺗﻔﻜﺮ و ﺗﺸﻌﺮ. أرﺑﻂ ﺑﻴﻦ إﺟﺎﺑﺘﻚ و أﻓﻜﺎرك و ﻣﺸﺎﻋﺮك اﻟﺤﺎﻟﻴﺔ. و رﺑﻤﺎ ﺗﺘﻌﻠﻖ ﺑﻌﺾ اﻷﺳﺌﻠﺔ ﺑﺤﻴﺎﺗﻚ اﻟﺨﺎﺻﺔ، إﻻ أﻧﻬﺎ ﺿﺮورﻳﺔ ﻟﻘﻴﺎس آﺎﻓﺔ ﺟﻮاﻧﺐ

ﺣﻴﺎﺗﻚ ﻋﻠﻰ ﻧﺤﻮ آﺎف.

**ﻳﺮﺟﻰ اﺳﺘﻜﻤﺎل اﻟﺒﻨﻮد اﻟﻤُﻌﻠﻤﺔ ﺑﺎﻟﻌﻼﻣﺔ اﻟﻨﺠﻤﻴﺔ (*) ﻓﻘﻂ إذا آﺎن ﻟﺪﻳﻚ ﺷﺮﻳﻚ ﺣﻴﺎة.**

| **ﺟﻴﺪ ﺟﺪا** | **ﺟﻴﺪ** | **ﻻ ﺟﻴﺪ و ﻻ ﺳﻴﺊ** | **ﺳﻴﺊ** | **ﺳﻴﺊ ﻟﻠﻐﺎﻳﺔ** | **و ﻟﻜﻞ ﺳﺆال، ﺗﺤﻘﻖ ﻣﻦ ﺗﻠﻚ اﻹﺟﺎﺑﺔ اﻟﺘﻲ ﺗﻜﻮن أﻗﺮب إﻟﻰ أﻓﻜﺎرك**  **و ﻣﺸﺎﻋﺮك اﻟﺤﺎﻟﻴﺔ** |  |
| --- | --- | --- | --- | --- | --- | --- |
| 🞏 | 🞏 | 🞏 | 🞏 | 🞏 | آﻴﻒ ﺗﻘﻴﻢ ﺻﺤﺘﻚ؟ | A |
| **راض ﺟﺪا** | **راض** | **ﻻ راض و ﻻ ﻏﻴﺮ راض** | **ﻏﻴﺮ راض** | **ﻏﻴﺮ راض ﻟﺤﺪ آﺒﻴﺮ** | **و ﻟﻜﻞ ﺳﺆال، ﺗﺤﻘﻖ ﻣﻦ ﺗﻠﻚ اﻹﺟﺎﺑﺔ اﻟﺘﻲ ﺗﻜﻮن أﻗﺮب إﻟﻰ أﻓﻜﺎرك**  **و ﻣﺸﺎﻋﺮك اﻟﺤﺎﻟﻴﺔ** |  |
| 🞏 | 🞏 | 🞏 | 🞏 | 🞏 | هﻞ أﻧﺖ راض ﻋﻦ ﻧﻮﻋﻴﺔ ﺣﻴﺎﺗﻚ؟ | B |
| **ﻻ إﻃﻼﻗﺎ** | **ﻟﻴﺲ آﺜﻴﺮا** | **ﺑﺎﻋﺘﺪال** | **ﻟﺤﺪ آﺒﻴﺮ** | **ﺗﻤﺎﻣﺎ** | **و ﻟﻜﻞ ﺳﺆال، ﺗﺤﻘﻖ ﻣﻦ ﺗﻠﻚ اﻹﺟﺎﺑﺔ اﻟﺘﻲ ﺗﻜﻮن أﻗﺮب إﻟﻰ أﻓﻜﺎرك**  **و ﻣﺸﺎﻋﺮك اﻟﺤﺎﻟﻴﺔ** |  |
| 🞏 | 🞏 | 🞏 | 🞏 | 🞏 | هﻞ ﻳُﻀﻌﻒ اﻟﺘﻔﻜﻴﺮ ﺑﺎﻟﻌﻘﻢ ﻣﻦ اﻧﺘﺒﺎهﻚ و ﺗﺮآﻴﺰك ؟ | 1 |
| 🞏 | 🞏 | 🞏 | 🞏 | 🞏 | هﻞ ﺗﻌﺘﻘﺪ ﺑﺄﻧﻪ ﻻ ﻳﻤﻜﻨﻚ اﻟﻤﻀﻲ ﻗﺪﻣﺎ ﻟﺘﺤﻘﻴﻖ أهﺪاف وﺧﻄﻂ ﺣﻴﺎﺗﻚ  اﻷﺧﺮى ﺑﺴﺒﺐ ﻣﺸﺎآﻞ اﻟﺨﺼﻮﺑﺔ؟ | 2 |
| 🞏 | 🞏 | 🞏 | 🞏 | 🞏 | هﻞ ﺗﺸﻌﺮ ﺑﺄﻧﻚ ﻣﺴﺘﻨﺰف اﻟﻄﺎﻗﺔ و ﻣﺮهﻖ ﺑﺴﺒﺐ ﻣﺸﺎآﻞ اﻟﺨﺼﻮﺑﺔ؟ | 3 |
| 🞏 | 🞏 | 🞏 | 🞏 | 🞏 | هﻞ ﺗﺸﻌﺮ ﺑﺄﻧﻚ ﻗﺎدرا ﻋﻠﻰ ﻣﻮاﺟﻬﺔ ﻣﺸﺎآﻠﻚ اﻟﻨﺎﺟﻤﺔ ﻋﻦ اﻟﺨﺼﻮﺑﺔ؟ | 4 |
| **راض ﺟﺪا** | **راض** | **ﻻ راض و ﻻ ﻏﻴﺮ راض** | **ﻏﻴﺮ راض** | **ﻏﻴﺮ راض ﻟﺤﺪ آﺒﻴﺮ** | **و ﻟﻜﻞ ﺳﺆال، ﺗﺤﻘﻖ ﻣﻦ ﺗﻠﻚ اﻹﺟﺎﺑﺔ اﻟﺘﻲ ﺗﻜﻮن أﻗﺮب إﻟﻰ أﻓﻜﺎرك**  **و ﻣﺸﺎﻋﺮك اﻟﺤﺎﻟﻴﺔ** |  |
| 🞏 | 🞏 | 🞏 | 🞏 | 🞏 | هﻞ أﻧﺖ راض ﻋﻦ اﻟﺪﻋﻢ اﻟﺬي ﺗﺘﻠﻘﺎﻩ ﻣﻦ أﺻﺪﻗﺎﺋﻚ ﻓﻴﻤﺎ ﻳﺘﻌﻠﻖ  ﺑﻤﺸﺎآﻞ اﻟﺨﺼﻮﺑﺔ ﻟﺪﻳﻚ؟ | 5 |
| 🞏 | 🞏 | 🞏 | 🞏 | 🞏 | هﻞ أﻧﺖ راض ﻋﻦ ﻋﻼﻗﺘﻚ اﻟﺠﻨﺴﻴﺔ ﻋﻠﻰ اﻟﺮﻏﻢ ﻣﻦ ﻣﺸﺎآﻞ  اﻟﺨﺼﻮﺑﺔ ﻟﺪﻳﻚ؟ | *6 |
| **أﺑﺪا** | **ﻧﺎدرا** | **ﻏﺎﻟﺒﺎ ﻣﺎ ﻳﻜﻮن** | **اﻟﻜﺜﻴﺮ ﻣﻦ اﻷﺣﻴﺎن** | **داﺋﻤﺎ** | **و ﻟﻜﻞ ﺳﺆال، ﺗﺤﻘﻖ ﻣﻦ ﺗﻠﻚ اﻹﺟﺎﺑﺔ اﻟﺘﻲ ﺗﻜﻮن أﻗﺮب إﻟﻰ أﻓﻜﺎرك**  **و ﻣﺸﺎﻋﺮك اﻟﺤﺎﻟﻴﺔ** |  |
| 🞏 | 🞏 | 🞏 | 🞏 | 🞏 | هﻞ ﺗﺴﺒﺐ ﻣﺸﺎآﻞ اﻟﺨﺼﻮﺑﺔ ﻟﺪﻳﻚ اﻟﺸﻌﻮر ﺑﺎﻟﻐﻴﺮة و اﻻﺳﺘﻴﺎء؟ | 7 |
| 🞏 | 🞏 | 🞏 | 🞏 | 🞏 | هﻞ ﺗﻌﺎﻧﻲ ﻣﻦ اﻟﺤﺰن و/أو اﻟﺸﻌﻮر ﺑﻔﻘﺪان ﺷﻴﺌﺎ ﻣﺎ ﺣﻮل ﻋﺪم ﻗﺪرﺗﻚ  ﻋﻠﻰ اﻟﺤﺼﻮل ﻋﻠﻰ ﻃﻔﻞ )أو اﻟﻤﺰﻳﺪ ﻣﻦ اﻷﻃﻔﺎل( | 8 |
| 🞏 | 🞏 | 🞏 | 🞏 | 🞏 | هﻞ ﺗﺘﺄرﺟﺢ ﻣﺸﺎﻋﺮك ﻣﺎ ﺑﻴﻦ اﻷﻣﻞ واﻟﻴﺄس ﺑﺴﺒﺐ ﻣﺸﺎآﻞ  اﻟﺨﺼﻮﺑﺔ؟ | 9 |
| 🞏 | 🞏 | 🞏 | 🞏 | 🞏 | هﻞ أﻧﺖ ﻣﻌﺰول اﺟﺘﻤﺎﻋﻴﺎ ﺑﺴﺒﺐ ﻣﺸﺎآﻞ اﻟﺨﺼﻮﺑﺔ؟ | 10 |
| 🞏 | 🞏 | 🞏 | 🞏 | 🞏 | هﻞ ﺗﺘﺒﺎدل أﻧﺖ و ﺷﺮﻳﻚ ﺣﻴﺎﺗﻚ ﻓﻴﻤﺎ ﺑﻴﻨﻜﻤﺎ اﻟﻤﺤﺒﺔ و اﻟﺤﻨﺎن ﺑﺎﻟﺮﻏﻢ  ﻣﻦ ﻣﺸﻜﻠﺔ اﻟﺨﺼﻮﺑﺔ ﻟﺪﻳﻚ؟ | *11 |
| 🞏 | 🞏 | 🞏 | 🞏 | 🞏 | هﻞ ﺗﺘﺪﺧﻞ ﻣﺸﺎآﻞ اﻟﺨﺼﻮﺑﺔ ﻟﺪﻳﻚ ﻣﻊ أﻋﻤﺎﻟﻚ اﻟﻴﻮﻣﻴﺔ و اﻟﺘﺰاﻣﺎﺗﻚ؟ | 12 |
| 🞏 | 🞏 | 🞏 | 🞏 | 🞏 | هﻞ ﺗﺸﻌﺮ ﺑﻌﺪم اﻻرﺗﻴﺎح ﻟﺤﻀﻮر اﻟﻤﻨﺎﺳﺒﺎت اﻻﺟﺘﻤﺎﻋﻴﺔ ﻣﺜﻞ  اﻹﺟﺎزات و اﻻﺣﺘﻔﺎﻻت ﺑﺴﺒﺐ ﻣﺸﺎآﻞ اﻟﺨﺼﻮﺑﺔ ﻟﺪﻳﻚ؟ | 13 |
| 🞏 | 🞏 | 🞏 | 🞏 | 🞏 | هﻞ ﺗﺸﻌﺮ ﺑﺄن ﺑﺈﻣﻜﺎن أﺳﺮﺗﻚ أن ﺗﺘﻔﻬﻢ ﻣﺎ ﺗﻤﺮ ﺑﻪ؟ | 14 |
| **ﻻ إﻃﻼﻗﺎ** | **ﻗﻠﻴﻼ** | **ﻗﺪر ﻣﻌﺘﺪل** | **آﺜﻴﺮ ﺟﺪا** | **أﻗﺼﻰ ﻗﺪر** | **و ﻟﻜﻞ ﺳﺆال، ﺗﺤﻘﻖ ﻣﻦ ﺗﻠﻚ اﻹﺟﺎﺑﺔ اﻟﺘﻲ ﺗﻜﻮن أﻗﺮب إﻟﻰ أﻓﻜﺎرك**  **و ﻣﺸﺎﻋﺮك اﻟﺤﺎﻟﻴﺔ** |  |
| 🞏 | 🞏 | 🞏 | 🞏 | 🞏 | هﻞ ﻋﺰزت ﻣﺸﺎآﻞ اﻟﺨﺼﻮﺑﺔ ﻟﺪﻳﻚ اﻟﺘﺰاﻣﻚ ﻟﺸﺮﻳﻚ ﺣﻴﺎﺗﻚ؟ | *15 |
| 🞏 | 🞏 | 🞏 | 🞏 | 🞏 | هﻞ ﺗﺸﻌﺮ ﺑﺎﻟﺤﺰن و اﻻآﺘﺌﺎب ﺣﻮل ﻣﺸﺎآﻞ اﻟﺨﺼﻮﺑﺔ ﻟﺪﻳﻚ؟ | 16 |
| 🞏 | 🞏 | 🞏 | 🞏 | 🞏 | هﻞ ﻣﺸﺎآﻞ اﻟﺨﺼﻮﺑﺔ ﻟﺪﻳﻚ ﺗﺠﻌﻠﻚ ﺗﺸﻌﺮ ﺑﺄﻧﻚ أﻗﻞ ﺷﺄن ﻣﻦ اﻟﻨﺎس  اﻟﺬﻳﻦ ﻣﻌﻬﻢ أﻃﻔﺎل؟ | 17 |
| 🞏 | 🞏 | 🞏 | 🞏 | 🞏 | هﻞ ﻳﺰﻋﺠﻚ اﻟﺘﻌﺐ ﺑﺴﺒﺐ ﻣﺸﺎآﻞ اﻟﺨﺼﻮﺑﺔ؟ | 18 |
| 🞏 | 🞏 | 🞏 | 🞏 | 🞏 | هﻞ ﻟﻤﺸﺎآﻞ اﻟﺨﺼﻮﺑﺔ ﺗﺄﺛﻴﺮ ﺳﻠﺒﻲ ﻋﻠﻰ ﻋﻼﻗﺘﻚ ﺑﺸﺮﻳﻚ ﺣﻴﺎﺗﻚ؟ | *19 |
| 🞏 | 🞏 | 🞏 | 🞏 | 🞏 | هﻞ ﺗﺠﺪ ﺻﻌﻮﺑﺔ ﻓﻲ اﻟﺘﺤﺪث ﻣﻊ ﺷﺮﻳﻚ ﺣﻴﺎﺗﻚ ﻋﻦ ﻣﺸﺎﻋﺮك  اﻟﻤﺘﻌﻠﻘﺔ ﺑﺎﻟﻌﻘﻢ؟ | *20 |
| 🞏 | 🞏 | 🞏 | 🞏 | 🞏 | هﻞ أﻧﺖ ﻣﺮﺗﺎح ﻣﻦ ﻋﻼﻗﺘﻚ ﺑﺎﻟﺮﻏﻢ ﻣﻦ ﻣﺸﺎآﻞ اﻟﺨﺼﻮﺑﺔ ﻟﺪﻳﻚ؟ | *21 |
| 🞏 | 🞏 | 🞏 | 🞏 | 🞏 | هﻞ ﺗﺸﻌﺮ ﺑﻀﻐﻂ اﺟﺘﻤﺎﻋﻲ ﻋﻠﻴﻚ ﻷن ﻳﻜﻮن ﻟﺪﻳﻚ أﻃﻔﺎل )أو اﻟﻤﺰﻳﺪ  ﻣﻦ اﻷﻃﻔﺎل(؟ | 22 |
| 🞏 | 🞏 | 🞏 | 🞏 | 🞏 | هﻞ ﺗﺆدي ﻣﺸﺎآﻞ اﻟﺨﺼﻮﺑﺔ ﻟﺪﻳﻚ إﻟﻲ ﺷﻌﻮرك ﺑﺎﻟﻐﻀﺐ؟ | 23 |
| 🞏 | 🞏 | 🞏 | 🞏 | 🞏 | هﻞ ﺗﺸﻌﺮ ﺑﺎﻷﻟﻢ وﻋﺪم اﻟﺮاﺣﺔ اﻟﺒﺪﻧﻴﺔ ﺑﺴﺒﺐ ﻣﺸﺎآﻞ اﻟﺨﺼﻮﺑﺔ ﻟﺪﻳﻚ؟ | 24 |

© European Society of Human Reproduction & Embryology and American Society of Reproductive Medicine

**English and Arabic FertiQoL interview topic guide**

- Provide information about quality of life (QoL) in general (simplified version of the WHO definition) 🡪 “Quality of life (**QOL**) is the general well-being of individuals and societies, outlining negative and positive features of life. It observes life satisfaction, including everything from physical health, family, education, employment, wealth, religious beliefs and the environment.”

نوعية الحياة: الرفاه العام للأفراد والمجتمعات، و تتضمن السمات السلبية والإيجابية للحياة. الرضا عن الحياة، بما في ذلك كل شيء من الصحة البدنية والأسرة والتعليم والعمل والثروة، والمعتقدات الدينية، والبيئة.

- Ask the open-ended question: How has infertility impacted your life?

كيف أثرت مشاكل الخصوبة في حياتك؟

- Can prompt further with: What are the important areas of your life that infertility has impacted positively or negatively?

ما هي النطاقات الهامة في حياتك التي تأثر سلبا أو إيجابا من مشاكل الخصوبة؟

- Administer the FertiQol & reassure the participants that there are no right or wrong answers, that we are only interested in *their* experiences.

طمأن المشارك أنه لا توجد إجابة صحيحة أو خاطئة، أننا مهتمون فقط في تجاربهم

- Ask about extreme scores on ITEMs – ‘you scored x on this item, please would you tell me more about why you scored the item in this way

على هذا البند x أنت أجبت

سبب أجابتك بهذه الطريقة هل يمكن أن تخبرني المزيد عن

- Score FertiQoL online, show them the chart (explain the 0-100 scale, use analogy) and ask about the domains – does this reflect your experience?’ and then ask about contrasting of the domains: ‘we can see that your scores are not equal across domains, does this reflect your experience?’

هل هذا يعكس تجربتك

يمكننا أن نرى أن علاماتك ليست متساوية عبر المجالات، فهل هذا يعكس تجربتك؟

**Arabic debrief**

**شرح الغرض من المقابلة والاستبيانات**

واحدة من أهم القضايا في تحديد الصحة هي الطريقة التي ننظر بها لصحتنا و الأمراض. وقد استخدمت حملات الصحة العامة الناجحة استراتيجية لزيادة الوعي عن بعض الأمراض عن طريق البحث في المؤشرات الصحية المناسبة لكل مرض، وضمان أن معظم الناس يدركون علامات وأعراض الأمراض (مثل السرطان وأمراض القلب). وقد أبرزت هذه البحوث أن هذا النهج يمكن استخدامه لرصد احتياجات الرعاية الصحية، وتقييم مدى فعالية وتأثير برامج الرعاية الصحية.

بالنسبة لغالبية الأزواج يحدث الحمل بعد المحاولة لمدة اثني عشر شهرا. ومع ذلك، لعدد من الأزواج قد يستغرق وقتا أطول. هناك القليل من الأبحاث عن المؤشرات الرئيسية لتأخر الحمل. وبالإضافة إلى ذلك قلة من الناس تعرف علامات المرض التناسلي أو عوامل صعوبات الخصوبة. وقد تم تطوير أداة لرفع مستوى الوعي حول عوامل الخطر لمشاكل الخصوبة وتزويد النساء بالمعلومات حول ما يجب القيام به عندما يكون لديهم أي مخاطر. نحن أيضا قمنا بتطوير أداة/استبيان حول نوعية الحياة يسمى FertiQoL. ومع ذلك، فإننا لا نعرف ما إذا كانت هذه الأدوات يمكن أن تستخدم في بلدان غير حيث تم وضعها.

كان من المهم طرح مجموعة من الأسئلة الشخصية حول نمط حياتك وتاريخ الإنجاب، ونود أن نؤكد لكم أن جميع البيانات التي قدمتها ستعقد مجهول، وأنه لن يكون من الممكن تتبع المعلومات مرة أخرى لك. سيتم تخزين البيانات على جهاز كمبيوتر (محمي بكلمة مرور) وينتمي إلى/المملوك لدى رشا بيومي والأستاذ جاكي بوافين.

إذا كان الاشتراك في هذه الدراسة قد تسبب في القلق بشأن صحتك الرجاء الاتصال بالطبيب بالطريقة المعتادة أو عن طريق صفحة الفيسبوك – استشارات أمراض النساء والتوليد و الخصوبة، التي توفر الدعم للنساء الذين يعانون من مشاكل الخصوبة.

إذا كان لديك أي أسئلة أخرى حول هذا البحث اسأل رشا بيومي أو طبيبك عن هذه المخاوف، وأنهم سوف إبلاغ الأستاذ جاكي بوافين.

كلية علم النفس

جامعة كارديف

برج البناء، بارك بليس

كارديف، ويلز

CF10 3AT

boivin@cardiff.ac.uk

أستاذة جاكي بوافين تهتم بالجوانب النفسية والاجتماعية للصحة الإنجابية. وقالت إنها أجرت العديد من الدراسات في هذا المجال على قضايا مثل العلاقة بين التوتر والخصوبة، والاختلافات بين الرجال والنساء في ردود الفعل العاطفية لقضايا الخصوبة، إذا الإرشاد يساعد الناس على التكيف مع مشاكل الخصوبة، نمواطفال الانابيب، والكثير. وقد تم تنفيذ هذا البحث مع مساعدة نساء من العديد من البلدان في جميع أنحاء العالم.

تفاصيل لجنة الأخلاق بكلية علم النفس:

البريد الإلكتروني: psychethics@cf.ac.uk

الهاتف: +44 (0) 29 208 74007 فاكس: +44 (0) 29 2087 4858. العنوان: أمين سر لجنة الأخلاق بكلية علم النفس.
